# The Role of 5-HT1A and 5-HT7 Receptors in Anxiety-like Behavior in Mice Treated with Subchronic Atorvastatin

**DOI:** 10.3390/cimb48090962

**Published:** 2026-09-20

**Authors:** Judith Espinosa-Raya, Alejandro Trejo-Lazaro, Alfredo Briones-Aranda, Rodrigo Romero-Nava, Fengyang Huang, Refugio Cruz-Trujillo, Josué Vidal Espinosa-Juárez

**Affiliations:** 1 Laboratorio Multidisciplinario en Ciencias Biomédicas, Escuela Superior de Medicina, Instituto Politécnico Nacional, Ciudad de Mexico 11340, Mexico; jespinosara@ipn.mx; 2 Pharmacology Laboratory, Faculty of Human Medicine, Autonomous University of Chiapas, Tuxtla Gutiérrez 29000, Mexico; alfredo.briones@unach.com.mx; 3 Laboratorio de Investigación en Genética de Enfermedades Metabólicas, Escuela Superior de Medicina, Instituto Politécnico Nacional, Ciudad de Mexico 11340, Mexico; 4 Obesity and Asthma Research Laboratory, Children’s Hospital of Mexico Federico Gomez, Mexico City 06720, Mexico; fhuang@himfg.edu.mx; 5 Químico Farmacobiólogo Program, Research Group on Health and Specialized Metabolites with Pharmacological Potential, Universidad Pablo Guardado Chávez (UPGCH), Libramiento Norte Oriente No. 3450, Tuxtla Gutiérrez 29040, Mexico; refugio.cruz@unach.mx (R.C.-T.); josue.espinosa@unach.mx (J.V.E.-J.); 6 Escuela de Ciencias Químicas, Universidad Autónoma de Chiapas, Ocozocoautla de Espinosa 29140, Mexico

**Keywords:** polypharmacology, 8-OH-DPAT, WAY100635, 5-HT1A receptor, 5-HT7 receptor, anxiety-like behavior

## Abstract

The polypharmacology of statins includes indirect interactions with the serotonergic system, particularly with the 5-HT1A and 5-HT7 receptors. Statins influence both the expression and function of these receptors, which may affect the regulation of anxiety. The main objective of this study was to examine the effects of subchronic treatment with atorvastatin (STA) and acute treatment with serotonergic drugs on anxiety-like behavior, as well as their possible relationship to changes in the expression of 5-HT1AR and 5-HT7R in different brain regions of mice. In the first phase, the effect of STA (10, 20, or 30 mg/kg daily for 21 days) on anxiety-related exploratory behavior was assessed in male CD1 mice using the avoidance exploratory behavior test. Concurrently, receptor expression levels were measured in the prefrontal cortex, hippocampus, and striatum following STA administration. In the second phase, the effect of serotonergic drugs (0.5 or 1 mg/kg of 8-OH-DPAT, a 5-HT1A/5-HT7 receptor agonist, or WAY100635, a 5-HT1A receptor antagonist) on anxiety-related behaviors was studied in separate groups of animals, stratified by dose, that had previously been treated with or without STA. The persistence of the anxiogenic effect following serotonergic drug administration in mice previously treated with STA may be related to the increased mRNA expression of 5-HT1AR and 5-HT7R observed in the hippocampus, striatum, and prefrontal cortex, although confirmation at the protein level is needed in future studies.

## 1. Introduction

Several neurotransmitter systems are involved in the regulation of anxiety, including the serotonergic, GABAergic, and dopaminergic systems [1]. The serotonergic system consists of neuronal pathways originating in the raphe nuclei and projecting to other brain regions such as the hippocampus, striatum, amygdala, and prefrontal cortex [2], all of which express high densities of 5-HT1A (5-HT1AR) and 5-HT7 (5-HT7R) receptors [3,4,5]. Experimental evidence indicates that a general decrease in 5-HT1AR expression or function is associated with anxiogenic-like behavior in mouse models, whereas its pharmacological activation produces anxiolytic-like effects [6,7]. However, the literature also reports contradictory outcomes depending on the brain region and behavioral paradigm. For example, hippocampal injection of 8-hydroxy-2-(di-n-propylamino)-tetralin (8-OH-DPAT) produces anxiolytic-like effects in the open field test and Vogel conflict test [8,9], although more complex and likely anxiogenic effects of hippocampal 8-OH-DPAT have been reported in the elevated plus maze [10,11].

Similarly, the role of the 5-HT7R in anxiety is not fully defined. Although previous studies have associated inactivation of the 5-HT7R with anxiogenic-like effects in mice evaluated in the avoidance exploratory behavior test [12], other studies have reported that administration of either a 5-HT7R agonist [13] or antagonist produces anxiolytic-like effects in the open field test [14,15,16]. These inconsistencies suggest that 5-HT7R-mediated modulation of anxiety is highly context-dependent.

Importantly, recent evidence indicates that 5-HT1AR and 5-HT7R interact functionally. Drugs such as HBK-15, a dual 5-HT1AR/5-HT7R antagonist [17], and lurasidone, which acts as a partial 5-HT1AR agonist and 5-HT7R antagonist [18], have been associated with anxiolytic effects in rodents, supporting the notion that these receptors function in concert.

Furthermore, there are discrepancies regarding possible neuropsychiatric side effects induced by statins, particularly their association with anxiety; such controversies justify this study. On the one hand, statins exert antioxidant, anti-inflammatory, and neuroplasticity-promoting effects associated with anxiolytic-like behaviors in clinical and preclinical studies [19,20]. On the other hand, this contrasts with evidence that statin-induced reduction in brain cholesterol decreases the activity of the serotonin reuptake transporter [21,22] and attenuates ligand binding to the 5-HT1AR [23,24], leading to anxiety and aggressive and impulsive behaviors [24].

Subchronic treatment with atorvastatin (STA) in mouse models has demonstrated modulatory effects on anxiety, although the outcomes appear to depend on the experimental context, as well as on age-related, statin-mediated differential processes of neurogenesis and angiogenesis in the animals. In adult mice, atorvastatin administration for six weeks, either alone or combined with an antihypertensive agent, was associated with accelerated fear-memory extinction, suggesting a possible facilitation of anxiety-reducing processes. However, in middle-aged mice, the combination of atorvastatin and captopril produced an anxiolytic-like effect in the open field test, whereas STA alone did not produce a significant effect in young mice [25].

In rat models of diet-induced hyperhomocysteinemia, STA (3 mg/kg/day for 30 days) produced an anxiolytic-like effect in animals evaluated in both the elevated plus-maze (EPM) and the open field test [20]. In contrast, subchronic oral administration of high doses of desimvastatin (88 mg/kg) or rosuvastatin (150 mg/kg) for 18 days induced an anxiogenic-like effect in rats evaluated in the EPM. Additionally, in a guinea pig study, oral treatment with simvastatin and atorvastatin for six weeks was associated with increased time spent swimming in the peripheral zone of the Morris water maze and higher swimming speed, behavioral changes interpreted as indicative of an anxiogenic-like effect [26]. Despite these reports, the neuropharmacological mechanisms underlying the anxiety-related effects of atorvastatin remain poorly understood, and the specific involvement of serotonergic receptor subtypes has not been clearly established.

Therefore, further investigation into the role of 5-HT1A and 5-HT7 receptors may have dual therapeutic implications, as both targets may be susceptible to STA treatment and are key components in the regulation of anxiety—an area in which studies conducted under these specific conditions remain scarce. Research employing selective serotonergic agents could thus contribute to a better understanding of both implications.

Considering the foregoing, this study aimed to examine the effects of STA and acute treatment with serotonergic drugs on anxiety-like behavior, as well as their possible relationship to changes in the expression of 5-HT1AR and 5-HT7R in different brain regions of mice.

## 2. Materials and Methods

### 2.1. Drugs

Atorvastatin (Pfizer, Ciudad de Mexico, Mexico; 10, 20, and 30 mg·kg^−1^, p.o.) or saline was administered daily for 21 days [27,28]. The 5-HT1A/5-HT7R agonist 8-OH-DPAT (0.05 and 0.1 mg·kg^−1^, i.p., −20 min) and the 5-HT1AR antagonist WAY100635 (0.5 and 1 mg·kg^−1^, i.p., −20 min) were obtained from Sigma Chemical Co. (St. Louis, MO, USA) and dissolved in 0.5% aqueous sodium carboxymethylcellulose solution [29,30,31]. Doses and administration latencies were selected based on previous studies.

### 2.2. Animals

All procedures were carried out in accordance with the guidelines for animal experimentation established by the Official Mexican Standard (NOM-062-ZOO-1999). The experimental protocols were approved by the Research Committee of the Autonomous University of Chiapas (Chiapas, Mexico; Authorization Number 03/ECQ/RPR/069/22; date of approval: 10 October 2022). Adult male CD-1 mice (25–30 g) were housed in groups of three in transparent acrylic cages (36 cm × 18.5 cm × 24 cm, shoe-box style) with wood-chip bedding, food (Lab Rodent Diet 5001), and tap water available ad libitum. The colony room was maintained on a 12 h light/12 h dark cycle (lights on from 09:00 to 21:00 h at 79.9 lux or 0.12 W/m^2^) at 22 ± 1 °C.

### 2.3. Experimental Groups

**Figure 1:**
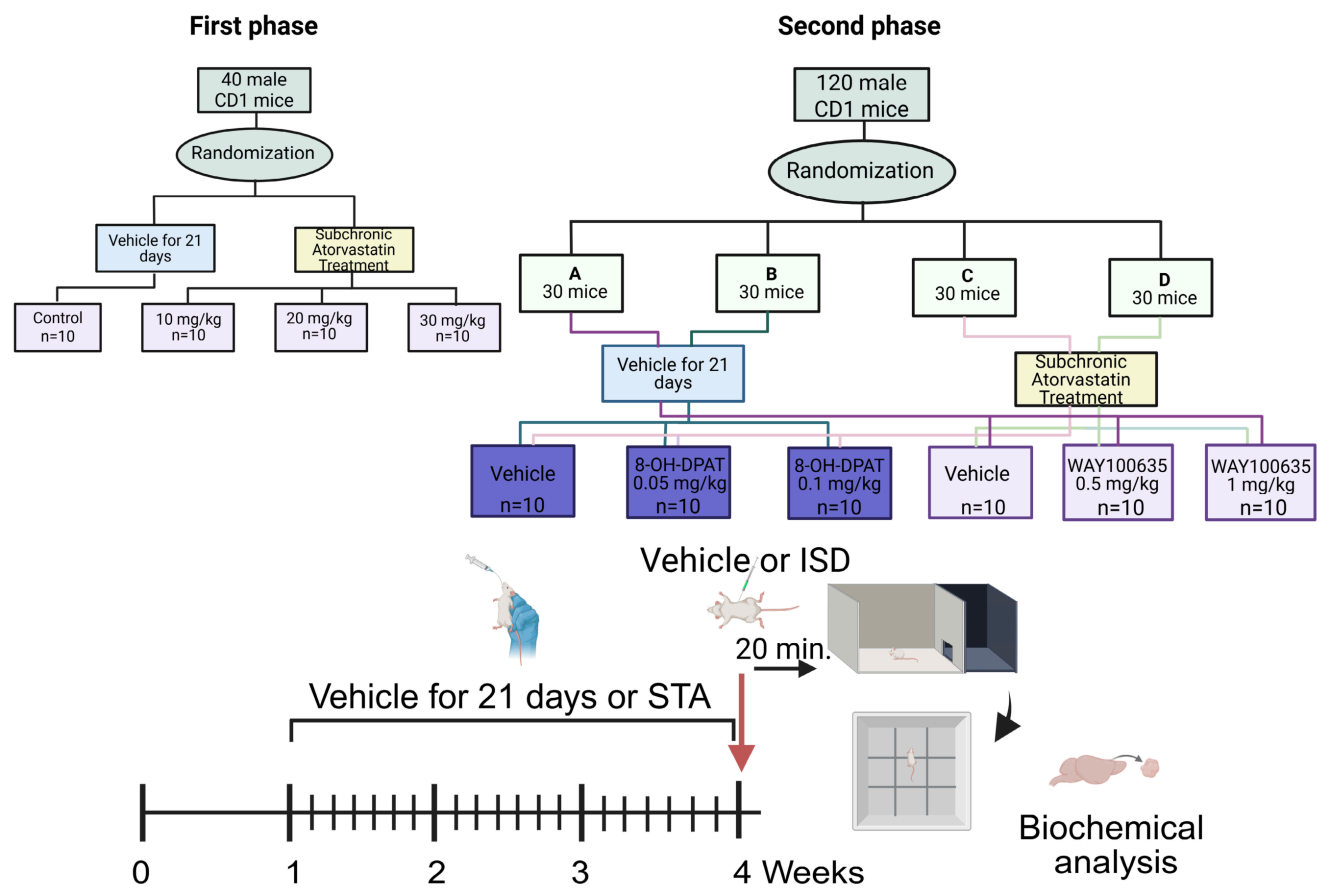
Flow diagram of the two experimental phases. The first phase describes the different subchronic treatments with atorvastatin (STA), and the second phase outlines the different groups of mice receiving acute serotonergic drug treatment either alone or in combination with STA (20 mg/kg). Experimental timeline: STA administration for 21 days, acute injection of serotonergic drugs (ISD), behavioral evaluation in the avoidance exploratory behavior test, followed by the open field test, sacrifice by decapitation, and brain dissection exclusively in the groups with and without STA for molecular analyses (Created in BioRender. Briones, A. (2026) https://BioRender.com/v27qwh3).

In the present study, sample size was determined using G*Power software (version 3.1.9.7). During the first phase, forty mice were randomly assigned to two groups: one comprising 10 animals and another comprising 30 animals. The first group received vehicle only (control group), while the second group was subdivided into three groups of 10 animals each, administered 10, 20, or 30 mg/kg of atorvastatin daily for 21 days, respectively (Figure 1).

In the second phase, a separate group of 120 mice was randomly divided into four groups of 30 animals each (A, B, C, and D). Group A was further subdivided into three subgroups of 10 animals according to dose: vehicle, 0.05 mg/kg, or 0.1 mg/kg of the 5-HT1A/5-HT7R agonist 8-OH-DPAT. Group B was subdivided and treated with the 5-HT1AR antagonist WAY100635 (0, 0.5, or 1 mg/kg). Groups C and D were also subdivided and treated following a mirror scheme of groups A and B, respectively (see colored lines in Figure 1), with the distinction that animals in both groups had previously received STA (20 mg/kg, p.o.). All groups were evaluated on day 22 in the avoidance exploratory behavior test and in the locomotor activity test and were subsequently sacrificed by decapitation (Figure 1).

### 2.4. The Avoidance Exploratory Behavior Test

This paradigm is a widely used procedure for studying experimental anxiety and screening drugs with potential anxiolytic activity. The apparatus consists of an acrylic cage (44 cm × 21 cm × 21 cm) divided into a small, darkened compartment (one-third of the total area) and a large, brightly illuminated compartment (560 lx; two-thirds of the total area), separated by a small opening (13 cm × 15 cm). In this test, each mouse was introduced (on a single occasion) into the illuminated compartment, and the number of transitions through the opening recorded over 10 min was used as an index of anxiety-like behavior [32].

### 2.5. Open Field Test

The open field test was used to evaluate locomotor activity in the mice comprising the different experimental groups [31]. The test was conducted in a black acrylic box (60 cm × 40 cm × 30 cm) with the floor divided into nine equal squares. Results were expressed as the total number of square crossings over 5 min. Each session was video recorded, and the apparatus was cleaned with 5% ethanol between subjects.

### 2.6. Tissue Preparation and RNA Isolation

Total RNA was isolated using TRIzol reagent (Life Technologies, Carlsbad, CA, USA) following the manufacturer’s instructions. Tissue samples were homogenized in TRIzol; chloroform was added for phase separation, and RNA was precipitated from the aqueous phase with isopropanol. The RNA pellet was washed with 75% ethanol, air-dried, and resuspended in RNase-free water. RNA concentration and purity were assessed spectrophotometrically by measuring absorbance at 260 and 280 nm. Samples were considered to have acceptable purity when the A260/A280 and A260/A230 ratios were approximately 1.8–2.0. RNA integrity was verified by electrophoresis on a 1% agarose gel, with distinct bands corresponding to 28S and 18S ribosomal RNA confirming sample integrity. The RNA pellet was dissolved in PCR-grade water and stored at −60 °C.

### 2.7. cDNA Synthesis

For cDNA synthesis, 1000 ng of total RNA from each sample was used as template. Reverse transcription was performed using M-MLV Reverse Transcriptase (Invitrogen, Carlsbad, CA, USA) according to the manufacturer’s protocol. The resulting cDNA was stored at −20 °C until use.

### 2.8. Primer Design and Real-Time Quantitative PCR

Specific primers were designed for the amplification of the genes of interest and the housekeeping gene (Table 1). Gene sequences were obtained from the NCBI GenBank database in FASTA format. Oligonucleotide design was performed using the online bioinformatics tool available at https://primers.neoformit.com/ (accessed on 2 July 2026). Given the use of SYBR Green detection chemistry, the following parameters were applied: primer length of 18–20 nucleotides, melting temperature (Tm) of 55–65 °C, GC content of 40–60%, and amplicon size of 80–150 base pairs. Primers were synthesized by Oligo T4 (Irapuato, Mexico). Gene expression was quantified by RT-qPCR using SYBR Green as an intercalating fluorescent dye. Reactions were prepared in a final volume of 10 µL containing 1× SYBR Green Master Mix, 0.3 µM of each primer (forward and reverse), cDNA equivalent to 1000 ng of initial RNA, and nuclease-free water. Amplification was performed using a MyGo Mini real-time PCR system (IT-IS Life Sciences) under the following thermal cycling conditions: initial denaturation at 95 °C for 10 min, followed by 45 cycles of 95 °C for 15 s and 60 °C for 30 s. A melting curve analysis (60–95 °C) with continuous fluorescence acquisition was subsequently performed to confirm amplification specificity, followed by a cooling step at 40 °C for 300 s.

### 2.9. Statistical Analyses

Statistical analyses were performed using SigmaPlot 12.0. Values are expressed as mean ± SEM. To evaluate normality, a Shapiro normality test was run on each experimental dataset. Potential outliers were identified using Grubbs’ test (alpha = 0.05); however, following this analysis, no values were identified that met the criteria for exclusion. One-way and two-way analyses of variance (ANOVA) were used to determine whether the treatments influenced anxiety-like behavior across the different groups. In the two-way ANOVA, STA was designated as factor A, while acute serotonergic drug treatment was designated as factor B. When significant differences were detected by ANOVA, group means were compared using Tukey’s post hoc test. The gene expression of 5-HT1AR and 5-HT7R in the brain regions examined was analyzed using Student’s *t*-test. A significance level of *p* < 0.05 was applied in all analyses.

## 3. Results

### 3.1. Effect of STA on Anxiety

**Figure 2:**
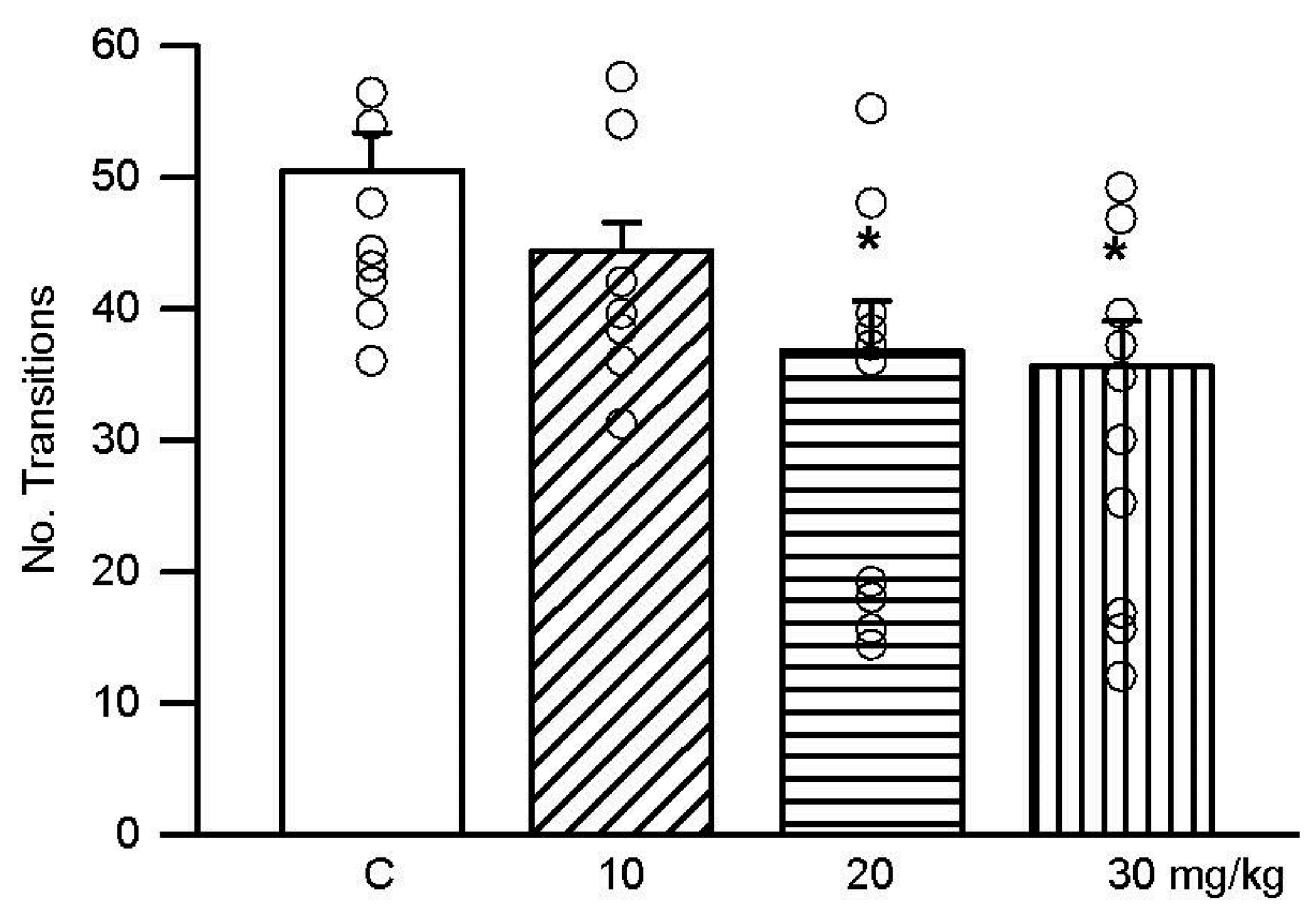
Effect of STA at different doses (10, 20, and 30 mg/kg) compared with its respective control group (C) on the number of transitions in the avoidance exploratory behavior test in male CD1 mice. Each bar (*n* = 10) represents the mean ± SEM. * *p* < 0.05 vs. control group (one-way ANOVA followed by Tukey’s test).

Figure 2 shows the number of transitions in the avoidance exploratory behavior test following STA treatment compared with the corresponding control group (F3,36 = 4.49; *p* < 0.05). A significant decrease in the total number of transitions was observed in mice treated with 20 or 30 mg/kg under the STA protocol. However, no significant difference in the number of transitions was found between these two groups. Therefore, only the 20 mg/kg dose was used in subsequent experiments.

**Figure 3:**
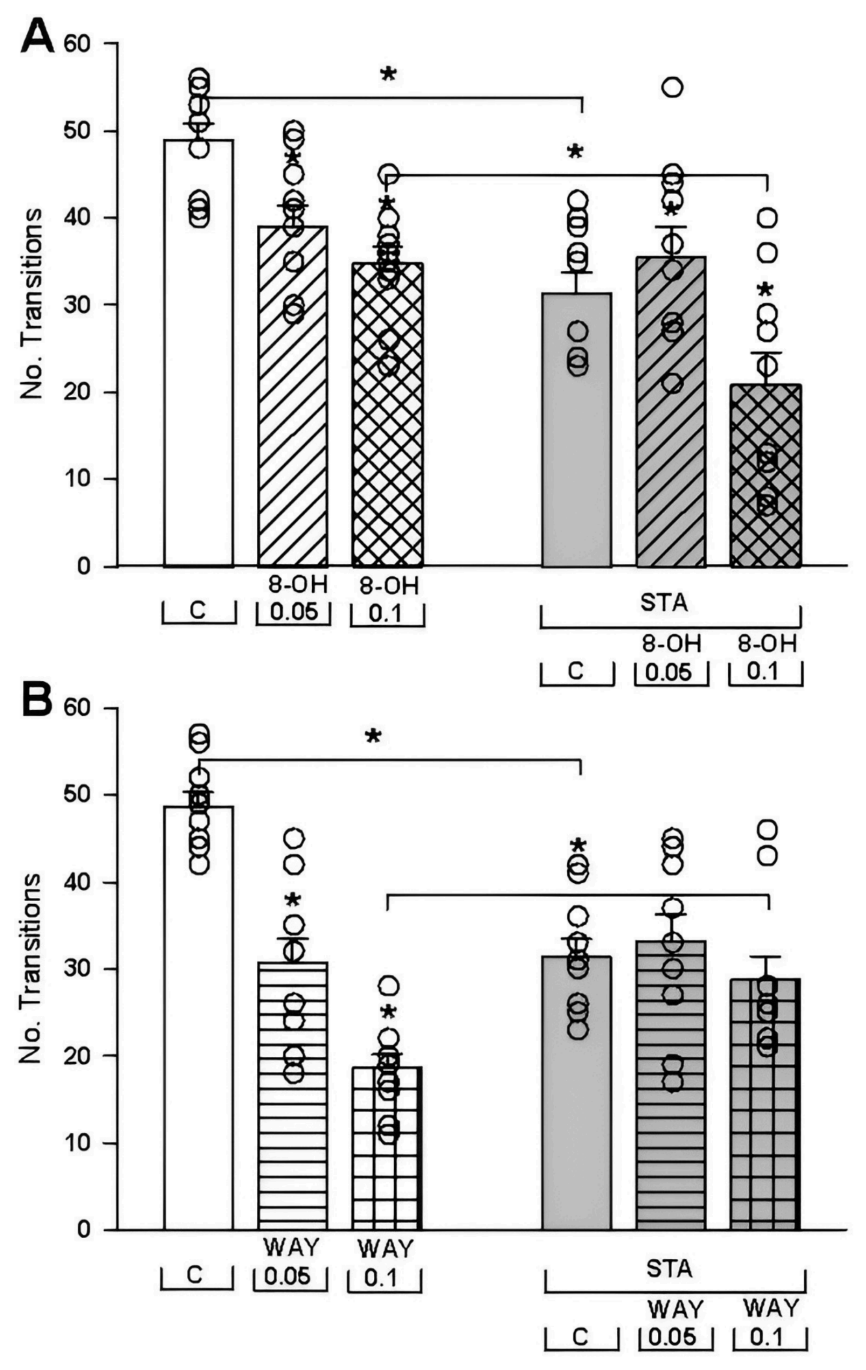
Effect of increasing doses of 8-OH-DPAT (0.05 and 0.1 mg/kg) (**panel A**) and WAY100635 (0.05 and 0.1 mg/kg) (**panel B**) on the number of transitions between the darkened and illuminated compartments in the avoidance exploratory behavior test. The effects of these drugs are compared between animals without and with subchronic treatment with atorvastatin (STA). Each bar (n = 10) represents the mean ± SEM. * *p* < 0.05 vs. respective control group (C) [(**panel A**), interaction A × B: F5,54 = 3.61; *p* < 0.05 and (**panel B**), interaction A × B: F5,54 = 17.89; *p* < 0.05 followed by Tukey’s test].

The locomotor activity data, expressed as the total number of squares crossed during a 5 min session, showed no statistically significant differences (see Table 2). Results are expressed as mean ± SEM. In the first panel, one-way analysis of variance revealed no significant differences [F(3,36) = 0.432; *p* > 0.05]. The different experimental groups treated with varying doses of serotonergic drugs in animals with and without prior STA are shown in Figure 1 under the designations A–C. The absence of significant differences in locomotor activity across all conditions confirms that the anxiety-like behavior observed (Figure 2 and Figure 3) was not confounded by this factor. Two-way analysis of variance was non-significant for both the second panel [interaction A × B: F(2,54) = 2.14; *p* > 0.05] and the third panel [interaction A × B: F(2,54) = 2.23; *p* > 0.05].

### 3.2. Influence of STA and the 5-HT1A Agonist on Anxiety

The number of transitions in the avoidance exploratory behavior test was dependent on both STA and acute administration of the 5-HT1A/5-HT7 receptor agonist. Post hoc comparisons revealed a significant decrease in the number of transitions (*p* < 0.05) in animals without prior STA treatment that received either dose of 8-OH-DPAT, compared with the vehicle group (Figure 3A).

A reduction in the number of transitions was also confirmed in the STA-plus-vehicle group compared with the corresponding STA-free control group. Furthermore, a decrease in the number of transitions was observed in mice treated with STA combined with acute 8-OH-DPAT (0.1 mg/kg), relative to both the STA plus vehicle group and the STA-free group receiving the same acute dose of 8-OH-DPAT (0.1 mg/kg).

### 3.3. Influence of STA and the 5-HT1A Antagonist on Anxiety

The results obtained with the WAY100635 protocol were similar to the pattern observed with the agonist 8-OH-DPAT. However, only in animals without prior STA treatment did both doses of the antagonist markedly reduce the number of transitions compared with the control group. In contrast, in STA-treated mice, neither dose of the antagonist altered the number of transitions relative to the STA plus vehicle group (Figure 3B). Notably, despite the absence of an antagonist effect under STA, the group receiving STA combined with WAY100635 (1 mg/kg) exhibited a significantly higher mean number of transitions compared with the STA-free group receiving the same dose (Figure 3B).

### 3.4. Effect of STA on 5-HT1AR and 5-HT7R Expression in Three Brain Regions

**Figure 4:**
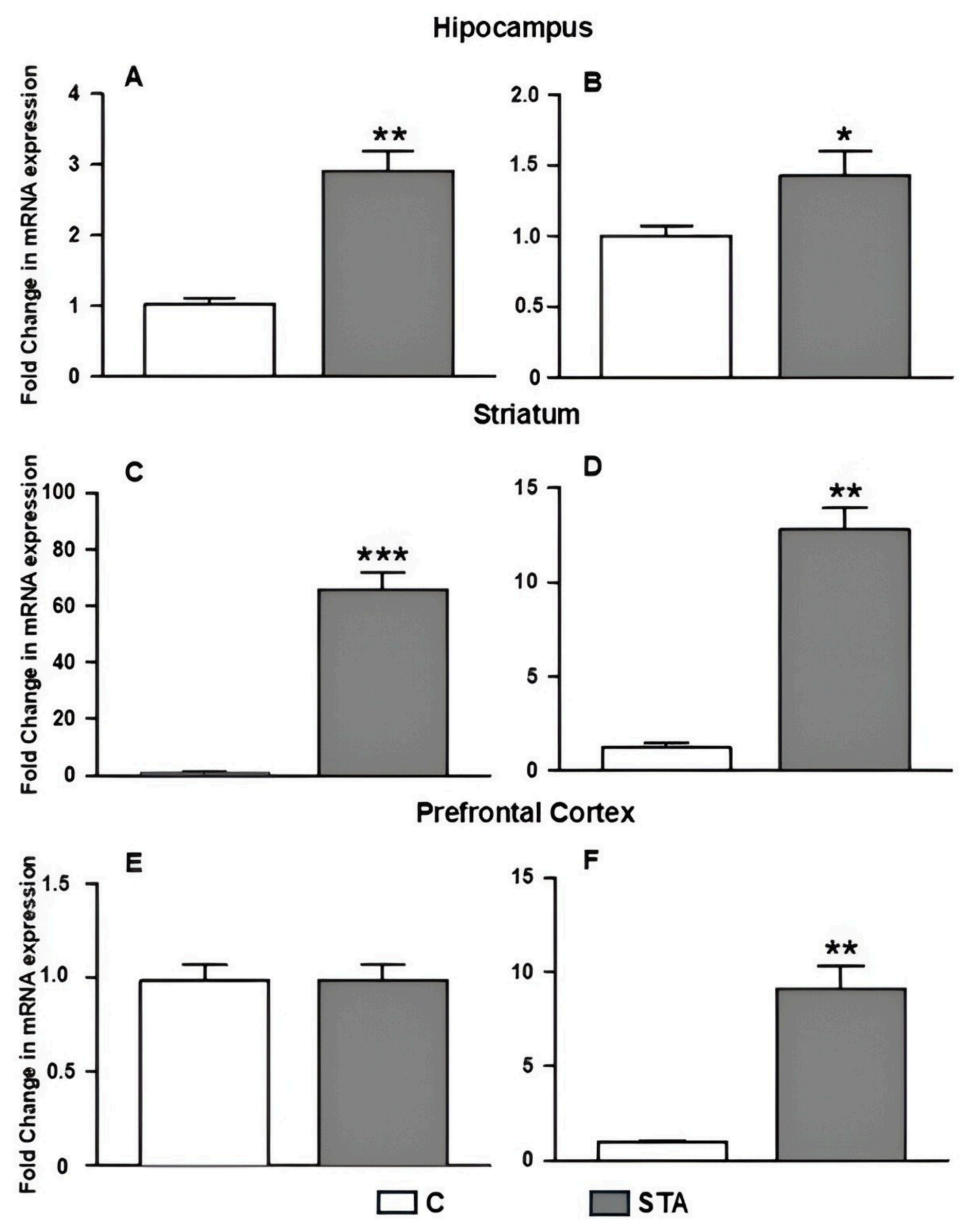
Gene expression of 5-HT1A (graphs on the left; (**A**,**C**,**E**)) and 5-HT7 (graphs on the right; (**B**,**D**,**F**)) serotonergic receptors in the hippocampus, striatum, and prefrontal cortex of mice following subchronic treatment with atorvastatin (STA), compared with their respective control groups (C). Data (*n* = 5) are expressed as mean ± SEM. The 36B4 gene was used as the reference gene. * *p* < 0.05, ** *p* < 0.01, and *** *p* < 0.001 vs. the respective control group.

STA significantly increased the mRNA expression of both 5-HT1AR and 5-HT7R relative to the reference gene 36b4 in the hippocampus, compared with the control group. Similarly, STA produced a significant increase in the expression levels of both receptors in the striatum. However, in the prefrontal cortex, this increase was observed only for 5-HT7R expression, as 5-HT1AR mRNA levels remained unchanged in this brain region (Figure 4).

## 4. Discussion

Given the growing interest in the behavioral effects of statins, the present study identified an anxiogenic-like effect following STA treatment, consistent with previous findings reporting a similar effect in mice evaluated in the EPM after STA treatment [33], as well as in rats evaluated in the EPM following subchronic administration of high doses of desimvastatin (88 mg/kg) or rosuvastatin (150 mg/kg) for 18 days [24]. An anxiogenic-like effect has also been reported in guinea pigs treated orally with simvastatin or atorvastatin for six weeks [26]. Similarly, four weeks of simvastatin administration (20 mg/kg) significantly attenuated the diazepam-induced anxiolytic-like effect in rats evaluated in the EPM [34].

In contrast, oral administration of simvastatin at 1–10 mg/kg for 21 days produced an anxiolytic-like effect in rats evaluated in the EPM [35]. However, other studies using high oral doses of atorvastatin (20–80 mg/kg) for 7 days reported no changes in anxiety levels in the EPM [36]. Taken together, the available evidence reveals inconsistent effects that may be attributed to several factors, including the type of statin, dosage, treatment duration, animal species, and behavioral model employed. Consistent with these discrepancies, clinical studies have reported a low risk of association between statin therapy and the prevalence of anxiety disorders [37], whereas other investigations have described an elevated risk of anxiety in adult statin users [38]. Therefore, continued experimental and clinical research is necessary to better understand these contrasting findings.

Regarding the anxiogenic-like effect that predominated following acute injection of either the agonist 8-OH-DPAT or the antagonist WAY100635 in mice with and without prior STA treatment, the behavioral effect produced by the agonist is consistent with previous reports. For example, administration of 8-OH-DPAT (0.25 mg/kg, i.p.) in rats evaluated using the same avoidance exploratory behavior test produced an anxiogenic-like effect, and 8-OH-DPAT was also shown to antagonize the anxiolytic effect induced by sodium nitroprusside [39]. Furthermore, administration of 8-OH-DPAT (1.0 mg/kg, s.c.) enhanced the anxiogenic-like effect in rats evaluated in the EPM [40].

In contrast, other studies have reported that subcutaneous injection of 8-OH-DPAT at 0.05–0.1 mg/kg produced anxiolytic-like effects in the EPM, while intraperitoneal administration at 0.1–1 mg/kg produced anxiolytic-like effects in the ultrasonic vocalization anxiety model [41]. Moreover, given that anxiety and impulsivity are distinct constructs, it has been shown that anxiogenesis can induce impulsivity. Consistent with this, subcutaneous 8-OH-DPAT (0.0125–0.1 mg/kg) increased impulsive action but decreased it at higher doses in rats evaluated in the novel paced variable consecutive number with discriminative stimulus task [42]. Biphasic effects of 8-OH-DPAT on anxiety markers and social behavior have also been reported [43].

The anxioselective profile of systemically administered WAY100635 has also been the subject of considerable interest, as studies conducted across different rodent anxiety paradigms have yielded a broad range of outcomes, ranging from anxiolysis [44,45] to absence of effect [46,47] to anxiogenesis [48]. This latter finding is consistent with the anxiogenic-like effect observed in the present study following administration of the 5-HT1AR antagonist WAY100635 in animals without prior STA treatment.

Furthermore, the high mRNA expression of 5-HT1AR and 5-HT7R observed in most of the brain regions analyzed in animals treated with STA reaffirms the role of statins in the serotonergic system, specifically in brain areas that are closely linked to the regulation of anxiety [3,4,5].

In another context, it is noteworthy that atorvastatin may promote increased internalization and degradation of 5-HT1AR, as well as a reduction in brain 5-HT levels, as reported in previous studies [49,50,51]. Furthermore, synaptosomal membrane cholesterol has been implicated in both the density of 5-HT receptors and the organization, dynamics, and function of the 5-HT1AR [24,49,52,53]; consequently, serotonergic dysfunction may occur.

**Figure 5:**
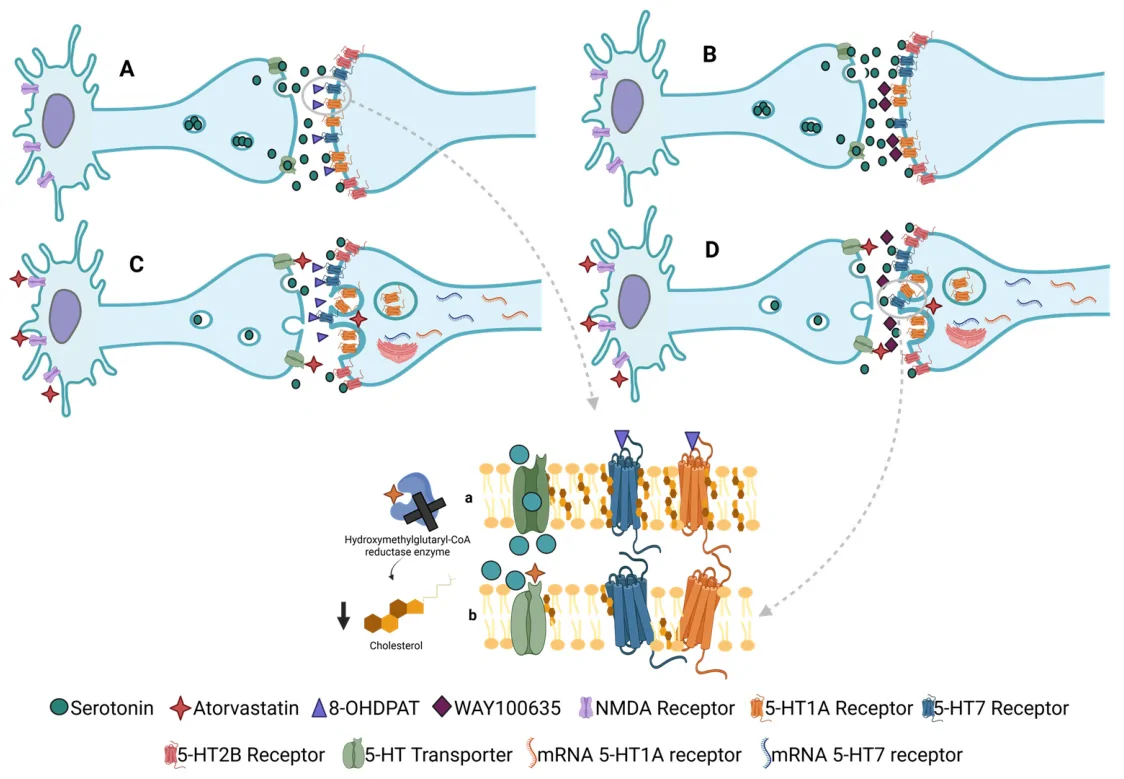
Hypothetical working model based on current findings and previous literature, illustrating the postsynaptic actions of the 5-HT1A/5-HT7R agonist 8-OH-DPAT (**A**) and the 5-HT1A receptor antagonist WAY100635 (**B**). The polypharmacology of atorvastatin may operate at multiple levels: NMDAR antagonism, inhibition of serotonin reuptake, and internalization and degradation of serotonergic receptors (**C**,**D**). STA may lead to reductions in both 5-HT levels and the number and functionality of serotonergic receptors, the latter mediated by decreased membrane cholesterol (a and b). These changes would favor the interaction of 8-OH-DPAT with 5-HT7R (**C**) and prevent antagonist binding to 5-HT1AR due to reduced availability of this target (**D**). Compensatory mechanisms, such as increased mRNA expression of both receptors, may account for the results observed (Created in BioRender. Briones, A. (2026) https://BioRender.com/jgkorpv).

Therefore, as a theoretical proposal, a hypothetical mechanistic model has been developed, which is described below (see Figure 5A–D). From this perspective, the ability of atorvastatin to modulate N-methyl-D-aspartate receptor (NMDAR) function has been demonstrated in vitro using neuroblastoma cells [54] and cortical neurons [55]. There is also substantial evidence that chronic administration of high doses of simvastatin in rats produced effects similar to those of NMDAR antagonists [35], suggesting that statins may exert specific antiexcitotoxic effects independent of 3-hydroxy-3-methylglutaryl-coenzyme A inhibition [55]. This is consistent with the role of NMDARs in the dorsal raphe nucleus in mediating inhibitory postsynaptic currents [56], suggesting that antagonism of this receptor could increase neuronal activity in the raphe nucleus [57,58,59], leading to enhanced 5-HT release in postsynaptic regions [60]. In support of this notion, phencyclidine, an NMDAR antagonist, has been shown to increase extracellular 5-HT levels in the prefrontal cortex and dorsal hippocampus [61,62]. It is therefore plausible that statins may enhance 5-HT release in postsynaptic regions through NMDAR antagonism. However, by promoting sustained 5-HT release, STA could ultimately deplete the stores of this indolamine.

Second, it is essential to consider the potential capacity of statins to interact with the serotonin transporter and reduce 5-HT reuptake [22,63,64]. Both premises—linking statins to NMDAR modulation and the reduction in 5-HT reuptake continuously favored by STA—reasonably support the hypothesis of decreased brain 5-HT levels (see Figure 5C,D). Consistent with this, mice exposed to chronic stress also exhibited an anxiogenic-like effect [65], and rats treated with simvastatin for 2 or 4 weeks spent more time in the open arms of the EPM—traditionally interpreted as an anxiolytic-like effect but reinterpreted in that study as increased impulsivity [64]. Therefore, the anxiogenic-like effect observed in the present study may be related to STA-induced changes in 5-HT levels, as proposed in Figure 5C,D.

Nonetheless, it is important to acknowledge the limitations of this study, which include the lack of measurement of relevant neurotransmitters, such as 5-HT; the absence of experiments with selective 5-HT7R ligands; the lack of additional 5-HT1A serotonergic drugs; the use of a single behavioral paradigm; and the absence of protein quantification of both receptors by western blotting. Furthermore, the functionality of the 5-HT1A and 5-HT7 receptors was not evaluated, nor were parallel experiments conducted in female rats; while the latter would have allowed for consideration of the influence of variations in estrogen levels on anxiety-related behavior, it would have made it more difficult to distinguish the effect of STA. Furthermore, it is recognized that the changes observed in mRNA expression do not constitute direct evidence of changes in the quantity or functionality of receptors. Nevertheless, addressing all these aspects would have strengthened the proposed conclusions, which require rigorous and gradual experimental development. Therefore, these limitations define the scope of the current study and point to relevant avenues for future research.

Furthermore, it is noteworthy that only the highest dose of 8-OH-DPAT significantly enhanced the anxiogenic-like effect in mice previously treated with STA. This finding is consistent with reports linking statin treatment to 5-HT1AR internalization [66], as well as with the affinity of 8-OH-DPAT for the 5-HT7R as an agonist [29,67]. Notably, 5-HT7R agonists have been associated with anxiogenic-like effects in mice [12]. Therefore, given a potential STA-induced reduction in 5-HT1AR availability, the proposed hypothesis is that 8-OH-DPAT would preferentially interact with the 5-HT7R, thereby producing an anxiogenic-like effect (Figure 5C).

Taken together, the effects produced by this agonist are complex to interpret, as 5-HT1AR—the primary target with which this drug interacts—can be expressed both pre- and postsynaptically and across multiple brain regions, where it may exert divergent effects on anxiety (Figure 5A,B).

It is therefore plausible to propose that blockade of 5-HT1ARs increases the likelihood that available 5-HT in the synaptic cleft interacts with other serotonergic receptors, such as 5-HT2AR and 5-HT7R—receptors that have been associated with anxiogenic-like behaviors [12,68] (Figure 5B). An alternative possibility is that WAY100635 may act as an inverse agonist at the 5-HT1AR, as has been demonstrated in a previous study [69].

Nevertheless, the apparently contradictory effects of the 5-HT1A antagonist on anxiety-like behavior further highlight the possibility of divergent outcomes depending on the experimental context, which may be influenced by multiple factors such as the rodent species used, prior pharmacological treatments, the dosing regimen employed, and the behavioral model utilized.

It is also important to consider that, although WAY100635 did not modify anxiety levels in mice previously treated with STA relative to the STA-alone group, the highest dose (1 mg/kg) did produce a significant increase in the number of transitions in STA-treated animals when compared with the corresponding mirror group that did not receive atorvastatin. This is interpreted as a partial attenuation of the anxiogenic-like effect that did not reach anxiolytic levels, demonstrating the complexity of the mechanisms involved. Nevertheless, it is important to acknowledge that further research is needed to consolidate these proposals.

## 5. Conclusions

The anxiogenic-like effect induced by STA is consistent with previous reports describing the ability of this statin to directly or indirectly regulate multiple components of the serotonergic system.

The enhancement and persistence of the anxiogenic-like effect produced by 8-OH-DPAT and WAY100635, respectively, in mice with prior STA treatment are consistent with the contrasting effects evidenced in previous studies and allude to both the influence of the experimental context and possible alterations in the serotonergic system.

The significant increase in mRNA expression of the 5-HT1A and 5-HT7 receptors in the hippocampus, striatum, and prefrontal cortex could highlight the role of statins in the serotonergic system located in brain areas that are closely linked to the regulation of anxiety, although confirmation at the protein level is required for future studies.

## Figures and Tables

**Table 1:** Sequences of primers used for qRT-PCR amplification.

Gene	Primer Left	Primer Right	ID GenBank
5HT7	GGGGGAAATCCTAACGCACA	TGCTATTGCCACACTCCCTG	NM_001347442.2
5HT1A	TGGGAGGAAGGGAGACTAGC	GGGGGAAGGGAGGGAAAAAG	NM_008308.5
Housekeeping Gene			
Gene	Primer Left	Primer Right	ID GenBank
36B4 (Rplp0)	CTGGAGAAACTGCTGCCTCA	CGCGCTTGTACCCATTGATG	NM_007475.5

**Table 2:** Effect of different pharmacological treatments on locomotor activity in mice, as assessed by the open field test.

Treatment (mg/kg)	Squares Crossed/5 min
Different subchronic treatments with atorvastatin (STA)	
Vehicle	20.88 ± 2.68
STA (10)	18.24 ± 2.50
STA (20)	16.27 ± 2.78
STA (30)	16.90 ± 2.78
Acute treatment with a 5-HT1A/5-HT7 agonist or in combination with STA	
Vehicle	18.97 ± 1.92
8-OH-DPAT (0.05)	20.08 ± 3.33
8-OH-DPAT (0.1)	16.65 ± 1.85
STA (20) mg/kg	16.27 ± 2.78
STA (20) + 8-OH-DPAT (0.05)	16.03 ± 2.52
STA (20) + 8-OH-DPAT (0.1)	18.90 ± 2.89
Acute treatment with a 5-HT1A antagonist or in combination with STA	
Vehicle	18.97 ± 1.92
WAY100635 (0.5)	19.97 ± 2.79
WAY100635 (1)	20.80 ± 4.02
STA (20)	16.27 ± 2.78
STA (20) + WAY100635 (0.5)	22.85 ± 2.29
STA (20) + WAY100635 (1)	24.09 ± 2.99

## Data Availability

The original contributions presented in this study are included in the article. Further inquiries can be directed to the corresponding authors.

## Abbreviations

The following abbreviations are used in this manuscript:

EPM	elevated plus-maze
5-HT1AR	5-HT1A receptor
5-HT7R	5-HT7 receptor
8-OHDPAT	8-Hydroxy-2-(din-propylamino)-tetraline
NMDAR	N-methyl-D-aspartate receptor
STA	subchronic treatment with atorvastatin
